# Molecular profiles in amygdala relevant to the relief of chronic unpredicted mild stress-induced depression by periodic meeting confidantes

**DOI:** 10.1093/scan/nsaf054

**Published:** 2025-05-23

**Authors:** Zhao Li, Jiaojiao Huang, Xiaoyu Chen, Lei Yao, Xianlong Zhao, Chang Liu, Hao Zhang, Zhenhua Song, Jin-hui Wang

**Affiliations:** Department of Pharmacology, Qingdao University, School of Pharmacy, Qingdao, Shandong, 266021, China; Department of Biology, College of Life Sciences, Qingdao Agricultural University, Qingdao, 266109, China; Department of Pharmacology, Qingdao University, School of Pharmacy, Qingdao, Shandong, 266021, China; School of Pharmacy, Yantai University, Yantai, 264005, China; Department of Pharmacology, Qingdao University, School of Pharmacy, Qingdao, Shandong, 266021, China; Department of Pharmacology, Qingdao University, School of Pharmacy, Qingdao, Shandong, 266021, China; Department of Pharmacology, Qingdao University, School of Pharmacy, Qingdao, Shandong, 266021, China; Department of Pharmacology, Qingdao University, School of Pharmacy, Qingdao, Shandong, 266021, China; College of Life Science, University of Chinese Academy of Sciences, Beijing, 100049, China

**Keywords:** depression, CUMS, confidantes, amygdala, microRNA

## Abstract

Social interaction with confidantes and living in groups are thought of as effective approaches to relieve affective disorders, especially major depression. The molecular mechanisms underlying this effectiveness remain largely unknown. Here, periodic interaction with confidante was used to study the effect of social support on depression-like behaviours induced by chronic unpredicted mild stress (CUMS), and high-throughput sequencing was used to analyse the miRNA and mRNA profiles in amygdala harvested from susceptible mice and resilience mice. The results showed that periodic interaction with confidante ameliorated CUMS-induced depression-like behaviours, and 194 differentially expressed genes (DEGs) were found to be associated with depression-like behaviours, 29 DEGs associated with resilience behaviours, and 152 DEGs associated with periodic meeting confidante. In addition, 98 differentially expressed microRNAs are associated with the relief of depression by confidantes. The microRNA-mRNA network associated with confidante-relieved depression has been established in the amygdala, based on our studies in microRNA and mRNA profiles. Taken together, our studies have revealed the potential new approach to improve depression-like behaviours induced by chronic stress, as well as many potential drug targets to prevent and treat major depression.

## Introduction

Major depressive disorder is the most prevalent mood disorder, which is characterized with depression, decreased volition, slow thinking, and cognitive decline (Camp and Cannon-Albright 2005, Battle 2013). In severe cases, suicidal behaviour may occur. According to epidemiological data, depression has become the leading cause of worldwide disability (Friedrich 2017). Since the onset of depression may be related to genetic, biological, and psychosocial factors, its pathogenesis is more complicated, and the mechanism is still unclear (Menard et al. 2016). Therefore, research on the pathogenesis and treatment of depression has attracted much attention.

Loss of close social connections and various other variables related to social relationships have been found to be important predictors of depression (Djernes 2006). A large number of studies have confirmed that few social relationships and low social support, especially low-perceived emotional support, are risk factors for depression (Blazer 2003, Tsai et al. 2005, Djernes 2006). In addition, studies have shown that loneliness can adversely affect the prognosis of depression, whether in young people or elderly people (van Beljouw et al. 2010, Holvast et al. 2015). Social support has been found to be very important to the course of depression, which raises the question of whether social support-related measures can be taken to intervene in depression. Although social support-related interventions have been used in the psychological treatment of depression, the molecular biological mechanism behind it is still unclear. Wang et al. discovered that having a companion can dramatically minimize the occurrence of depression-like behaviours using mouse model, and theorized that this could be due to the activation of mood and emotional reactions (An et al. 2020).

The amygdala in the limbic system plays a key role in regulating the mood and emotional reactions (Hastings et al. 2004, Garrett and Chang 2008), and is associated with mental health conditions such as social anxiety, posttraumatic stress disorder and depression. Studies suggest that acute stressors and chronic stress are strongly associated with neuronal activity within the amygdala (Correll et al. 2005). The basolateral part of the amygdala, which sends signals to the hippocampus or medial prefrontal cortex (mPFC), has shown that it is able to modulate social behaviours in a bidirectional manner (Felix-Ortiz and Tye 2014, Felix-Ortiz et al. 2016). In addition, the volume of the amygdala has also been shown to positively correlate with the number of social contacts and the number of social groups a person belongs to Bickart et al. (2011). Therefore, it will be very meaningful to test whether periodic interaction with a confidante helps to better prevent depression, and the molecular changes in the amygdala may provide better theoretical support for the application of social support in the treatment of depression.

In this study, chronic unpredictable mild stress (CUMS), a classic method for inducing depression-like behaviours, was used to treat mice, and periodic interaction with confidante was performed as a social support intervention. The miRNA and mRNA profiles of amygdala in susceptible mice and resilience mice were analysed by high-throughput sequencing to reveal the molecular changes. Our study is expected to reveal the molecular mechanisms underlying the effect of the periodic meeting confidantes on CUMS-induced depression, as well as to identify the molecules as effective potential targets for the prevention and treatment of major depression disorder.

## Materials and methods

### Mice

Three-week-old male C57BL/6J mice were purchased from Beijing Vital River Laboratory Animal Technology Co., Ltd, and raised in a controlled environment with free eating and drinking and light from 7 a.m. to 7 p.m. The temperature and relative humidity were kept at 22 ± 2°C and 55 ± 5%, respectively. The Qingdao University Animal Use and Care Committee approved the experimental protocol.

### CUMS processing and accompanying confidant treatment

**Figure 1.**
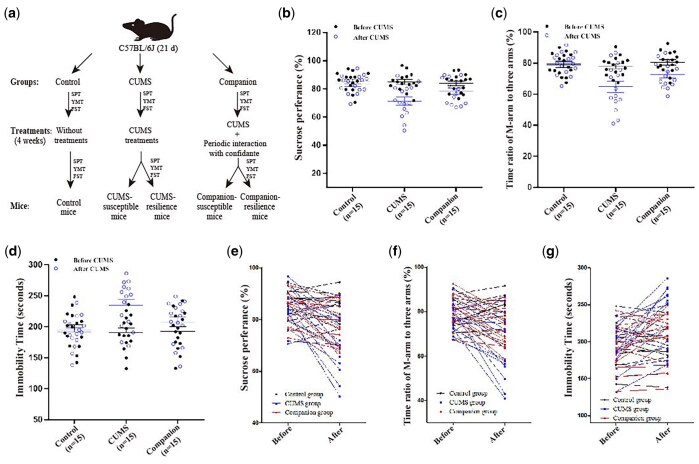
Periodic meeting confidante ameliorated depression-like behaviours induced by CUMS treatments. (a) The procedures to produce CUMS-susceptible, CUMS-resilience, Companion-susceptible, and Companion-resilience mice. These procedures including adaptation, behavioural tests, CUMS or periodic meeting confidante treatment, and group criteria based on behaviour. (b–d) The values of SPT, YMT and FST of mice in Control group (*n* = 15), CUMS group (*n* = 15) and Companion group (*n* = 15) before (black solid circle) and after (blue hollow circle) CUMS treatments. Two-way ANOVA was used for the comparisons among Control group, CUMS group and Companion group (Supplementary Table S3–S5). (e–g) Plot representative dots for SPT, YMT, and FST parameters, respectively.

The mice were kept for three days to adapt, and self-control data were collected by measuring sucrose preference test (SPT), Y-maze test (YMT), and forced swimming test (FST) before CUMS. Male mice were randomly divided into three groups based on behaviour test results: Control group (group housed without CUMS), CUMS group (with CUMS), and Companion group (treated by CUMS and accompanied by a companion) (Fig. 1a). As previously described, the CUMS program includes a variety of unpredictable mild stressors such as single cage, white noise, empty cage, damp sawdust cage, restraint space, strobe light, tilted cage, circadian disturbance, and social isolation (Supplementary Table S1) (Si et al. 2018, Sun et al. 2018, Shen et al. 2019, Guo et al. 2020). Except for the social isolation, these conditions were randomly selected to treat the mice in the manners of their separations or combinations every day (Xu et al. 2016). These treatments were applied for about 1-14 h in duration and 1-12 h in intervals (Xu et al. 2016). Mice in the Companion group were accompanied by a female confidant from the same litter for 30 min every three days when they were between two stressors (Fig. 1a and Supplementary Table S1).

### Behavioural definitions of susceptible and resilient mice

Behavioural tests were performed after CUMS or companion treatments to determine whether the mice experienced anhedonia and a lack of motivation. In order to measure anhedonia, indifference in a romantic partner, and self-esteem, the SPT, YMT, and FST are used (Porsolt et al. 1978, 1979, Willner et al. 1987, Strekalova et al. 2011, Overstreet 2012, Hao et al. 2019). According to our previous studies, the immobility time of the FST increased by 15% over its self-control values, while values in the SPT and YMT reduced by more than 20% of their self-control values, respectively, to identify susceptible mice (CUMS-susceptible animals in the CUMS group, Companion-susceptible mice in the Companion group). The behavioural criteria for CUMS-resilience mice in the CUMS group and Companion-resilience mice in the Companion group required that the range of changes in all three tests be within 5%.

### Extraction and purification of RNA

After defining susceptible and resilient mice based on behaviours, three mice per group were immediately anesthetized with isoflurane, perfused with normal saline in intra-heart, and decapitated. On an ice-cold glass slide, both sides of the entire amygdala region were separated. Total RNAs were isolated from amygdala using TRIzol Reagent (Life Technology, Carlsbad, CA, USA) as previously described (Si et al. 2018, Sun et al. 2018), and then three RNA samples per group were mixed together as one replicate. Three replicate samples per group were shipped to the Beijing Genomics Institute (BGI) in China for high-throughput sequencing analysis under dry ice conditions.

### Preparation of libraries, mRNA sequencing and data analysis

As previously reported, library preparation and mRNA sequencing were carried out as described in the following steps (Shen et al. 2019). Firstly, mRNAs were purified using Oligo (dT)-attached magnetic beads. Then, cDNA synthesis was performed, after which cDNA fragments were amplified using PCR and purified using Ampure XP beads. After being validated on the Agilent Technologies 2100 bioanalyzer, the qualifying library was amplified on cBot to produce the flow cell cluster, and the amplified flow cell was then sequenced single end on the HiSeq4000 platform.

The level of transcript expression was calculated using reads per kilobase per million mapped reads (RPKM). DEseq.2 was used to identify differentially expressed transcripts in samples. The |log (fold change, 2) |≥0.584936 and FDR ≤ 0.05 thresholds were used to identify differentially expressed genes (DEGs) when screening differentially expressed transcripts. DEGs were then annotated in the gene ontology (GO) database (http://www.geneontology.org/) and the Kyoto Encyclopedia of Genes and Genomes (KEGG) database (http://www.genome.jp/kegg).

### Preparation of libraries, miRNA sequencing and data analysis

As previously reported, library preparation and miRNA sequencing were carried out as described in the following steps (Shen et al. 2019). To prepare the library for each sample, 1 g total RNA was used. Total RNA was purified by electrophoretic separation on a 15% urea denaturing polyacrylamide gel electrophoresis (GAGE) gel, and small RNA regions corresponding to the marker lane’s 18–30 nt bands (14-30 ssRNA Ladder Marker, TAKARA) were excised and recovered. Small RNAs were transcribed into cDNA after adapter-ligation. The cDNA fragments were then enriched by PCR, separated by agarose gel electrophoresis with target fragments of 100-120 bp, and purified using the QIAquick Gel Extraction Kit (QIAGEN, Valencia, CA). Following quality assurance, the products were sequenced on the BGISEQ-500 platform.

To obtain clean reads, contaminated reads such as adapter dimers, junk, low complexity, and common RNA families (rRNA, tRNA, snRNA, and snoRNA) were removed. To identify known miRNAs, cleaned tags were annotated with miRBase 21.0. TPM values were used to calculate miRNA expression levels (Qiao et al. 2016). To compare known or novel miRNA expression among different groups, the DESeq software algorithm based on negative binomial distribution and biology duplicate samples was used. The fold-change greater than 1.5 and *P*-value .05 were used to identify the different expression of miRNAs. To identify the miRNA binding sites, three prediction methods (RNAhybrid, Targetscan, and miRanda) were used.

### Analysis of integrated miRNA/mRNA networks

The differentially expressed miRNA and transcript datasets were integrated using the following criteria: (1) In our study, miRNAs and mRNAs should undergo reverse changes at the same time; (2) The correlationship between miRNAs and their target mRNAs should be predicted by RNAhybrid, Targetscan, or miRanda software. The interactive network of differentially expressed miRNAs and concurrently expressed target mRNAs was visualized using Cytoscape software (San Diego, CA, USA).

### Quantitative RT-PCR for the validations of mRNA

To validate the results from High-throughput sequencing, we used quantitative real-time RT-PCR (qRT-PCR) by the SYBR Green technique to analyse mRNAs that were involved in different cellular functions, as well as were significantly different among Control mice (*n* = 3), CUMS-susceptible mice (*n* = 3), Companion-susceptible mice (*n* = 3), CUMS-resilience mice (*n* = 3), and Companion-resilience mice (*n* = 3), in which the samples were used from those tissues for high-throughput sequencing. Supplementary Table S2 lists the primers used in this study (Table S2). Briefly, RNAs were isolated from the tissues of the amygdala by using the Trizol method. cDNAs were synthesized for mRNA expression assays from total RNA in 10 μl volume with 2 μl PrimeScript™ RT Master Mix (TaKaRa Biotechnology Co. Ltd, Dalian, China), 1 μl total RNA and 7 μl ddH_2_O. qRT-PCR was done by using Biosystems QuantStudio 7 Flex (Life Technologies, USA). Each reaction was carried in a total volume of 20 μl, including 1 μl cDNA, 10 μl SYBR Premix Ex Taq™ II (TaKaRa Biotechnology Co. Ltd, Dalian, China), 0.5 μl of each primer (10 nmol/L), and 9 μl ddH_2_O, in which the program was set to 95°C in 5 min for pre-incubation, 40 cycles at 95°C in 5 s and at 60°C in 20 s for the annealing and amplification, as well as finally addition dissociation curve. The relative expression level of mRNAs in the tissue was normalized to an internal reference gene, Gapdh. All qRT-PCR runs were repeated in three replications. The results were calculated with the 2^-ΔΔCt^ method.

### Statistical analyses

The information from the behavioural tests was displayed as mean ± SEM. The control, CUMS, and Companion groups were compared statistically before and after treatment using a two-way ANOVA. Statistics of before versus after values within groups were compared using the paired *t*-test. The statistical comparison between control and CUMS-susceptible, control and CUMS-resilient, control and Companion-susceptible, control and Companion-resilient, CUMS-resilient and Companion-resilient, etc., were done using the unpaired Student *t*-test. *P *< .05 is considered statistically significant.

## Results

### Periodic meeting confidantes ameliorated depression-like behaviours induced by CUMS treatments

The CUMS model (Reid et al. 1997) was used to produce mice with depression-like behaviours, and periodic meeting confidantes was used to mimic a reward treatment. C57BL/6J male mice were divided into three groups, as shown in Fig. 1a: Control, CUMS and Companion. Mice in the CUMS and Companion groups were given CUMS for four weeks, with mice in the Companion group being accompanied by a confidante once every three days. The SPT, YMT, and FST were then used to assess depression-like behaviours or resilience behaviours; only mice who changed significantly in all three tests were classified as susceptible mice, and mice who did not change were classified as resilient mice. After 4 weeks of treatments, there were large variation in all three behavioural parameters of the CUMS and Companion groups. As shown in Fig. 1b–d, the SPT values (84.88 ± 1.666 versus 71.33 ± 2.908, *P *< 0.001, *n* = 15) (Fig. 1b and Supplementary Table S3) and the ratios of stay time in M-arm to stay time in total arms (78.04 ± 1.624 versus 64.77 ± 3.744, *P *< 0.001, *n* = 15) (Fig. 1c and Supplementary Table S4) significantly decreased, and the values of FST’s immobile time (190.7 ± 7.375 versus 234.5 ± 9.747, *P *< 0.001, *n* = 15) significantly increased (Fig. 1d and Supplementary Table S5) in CUMS group mice after 4 weeks treatments. These findings suggested that chronic CUMS treatment exposure caused mice to exhibit depression-like behaviours. In addition, pairwise comparisons between the three groups after CUMS revealed significant differences in SPT, YMT, and FST, respectively (Fig. 1b–d, Supplementary Tables S3–S5). Compared to the CUMS group, the SPT and YMT values significantly increased, and the values of FST’s immobile time significantly decreased in the Companion group (Fig. 1b–d, Supplementary Tables S3–S5). Furthermore, as shown in Supplementary Table S6, the percentage of susceptible mice in the CUMS group was approximately 33.33%, while it decreased to 20.00% in the Companion group. Meanwhile, the percentage of resilient mice in the CUMS group was around 13.33%, while it increased to 33.33% in the Companion group.

To precisely illustrate treatments-induced alterations, we plotted representative dots for SPT, YMT, and FST separately. As shown in Fig. 1e–g, for individual mice, CUMS treatments led to its behavioural changes. Nevertheless, when CUMS and periodic meeting confidantes were carried out concurrently, these alterations were minimized. These findings suggested that periodic meeting confidantes improved CUMS-induced depression-like behaviours in mice.

### Altered RNA expression profiles of amygdala in CUMS-susceptible, CUMS-resilience, companion-susceptible and companion-resilience mice compared to the control mice

**Figure 2.**
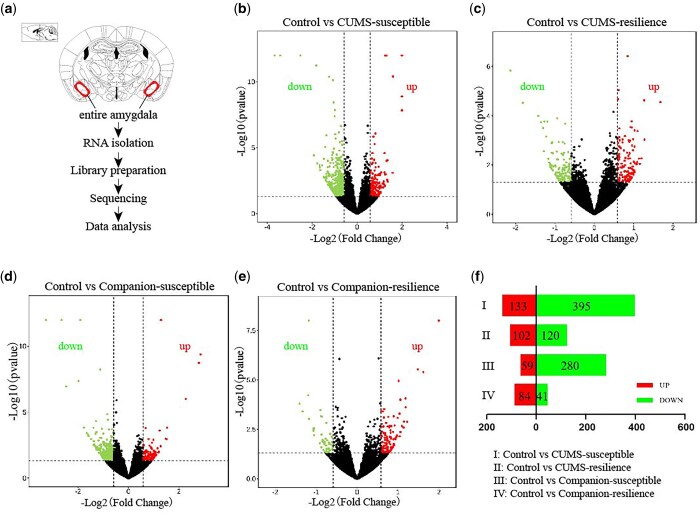
mRNA expression profiles of amygdala in induced susceptible and resilience mice. (a) Schematic diagram of the amygdala for samples collection and transcriptome sequencing. (b–e) The MA plots display the DEGs distribution between control and CUMS-susceptible mice, control and CUMS-resilience mice, control and companion-susceptible mice, control and companion-resilience mice, respectively. Up represents up-regulated genes; downs represent down-regulated genes. (f) The column chart shows the number of statistical DEGs.

To understand whether periodic meeting confidantes lead to changes at the molecular level in the amygdala region while ameliorating CUMS-induced depression-like behaviours, we used high-throughput sequencing to examine the mRNA profiles of amygdala regions collected from Control, CUMS-susceptible, Companion-susceptible, CUMS-resilience and Companion-resilience mice, and screened out DEGs (Fig. 2a). As shown in Fig. 2b–f and Supplementary Table S7, compared to the Control mice, there were 528 mRNAs differentially expressed in CUMS-susceptible mice, in which 133 mRNAs were up-regulated and 395 mRNAs were down-regulated. In Companion-susceptible mice, 339 DEGs were obtained, with 59 mRNAs up-regulated and 280 mRNAs down-regulated compared to the Control mice. In CUMS-resilience mice, 120 mRNAs were down-regulated, and 102 mRNAs were up-regulated compared to Control mice. In addition, 84 up-regulated mRNAs and 41 down-regulated mRNAs were obtained in Companion-resilience mice compared to the Control mice. These results indicated that the mRNA expression profiles of amygdala were altered, whether after CUMS treatments or CUMS with periodic meeting confidante treatments.

In order to validate our data above, we ran quantitative RT-PCR (qRT-PCR) from tissues that were used for mRNA sequencing. As shown in Supplementary Figs. S1–S4, the expressions of *Adora2a*, *Chrna6*, *Cldn11*, *Drd2*, *Ermn*, *Gabra2*, *Gabrq*, *Myo1d*, *Ntrk1*, *Slc12a2*, *Slc17a6* and *Slc17a8* in Control versus CUMS-susceptible, the expressions of *Camk2d*, *Chrna2*, *Gabra5*, *Glra2*, *Gng4*, *Grik4*, *Hap1*, *Nrp1*, *Nts*, *Scn5a*, and *Slc24a2* in Control versus CUMS-resilience, the expressions of *Cldn11*, *Daam2*, *Ermn*, *Gabra2*, *Lhx8*, *Plcl1*, *Slc6a9*, *Slc17a6*, and *Syt2* in Control versus Companion-susceptible, and the expressions of *Chrna4*, *Drd1*, *Glra2*, *Htr1d*, *Neurod2*, *Ntng2*, *Nts*, *Rgs9*, *Scn5a*, and *Syt6* in Control versus Companion-resilience were consistent with their sequencing data. Consistent results achieved by mRNA sequencing and qRT-PCR confirm the validation of our study.

### Identification of differentially expressed genes associated with depression-like behaviours in amygdala

**Figure 3.**
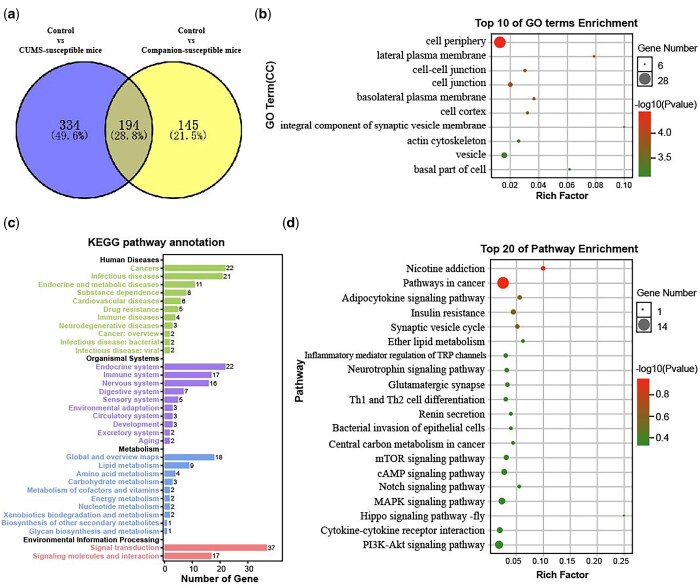
Functional analysis of DEGs associated with depression-like behaviours in amygdala. (a) Screening of DEGs associated with depression-like behaviours in Control versus CUMS-susceptible mice and Control versus Companion-susceptible mice through Venn diagram. (b) The histogram demonstrates common DEGs associated with depression-like behaviours GO enrichment results, CC (cellular component). (c) KEGG pathway annotation for common DEGs associated with depression-like behaviours. (d) The histogram demonstrates common DEGs associated with depression-like behaviours KEGG pathway enrichment results, top 20 of pathway enrichment were given.

According to consistent behavioural screening criteria, we obtained susceptible mice from the CUMS treated group and the Companion group, respectively. Therefore, if these two susceptible mice have common DEGs compared to control mice, these genes may be associated with depression-like behaviours. To test this, we analysed the DEGs of these two susceptible mice compared to the control mice and presented them using Venn diagrams. As shown in Fig. 3a, there were 194 common DEGs involved in Control versus CUMS-­susceptible and Control versus Companion-susceptible, and 334 DEGs particularly involved in Control versus CUMS-susceptible, as well as 145 DEGs particularly in Control versus Companion-­susceptible (Supplementary Table S8). Further analysis revealed that the expression patterns of the 194 common DEGs were consistent in both CUMS-susceptible and Companion-susceptible mice compared to control mice, whether up- or down-regulated (Supplementary Table S9).

To further explore the molecular information behind these 194 common DEGs associated with depression-like behaviours in the amygdala, GO and KEGG pathway enrichment analysis were carried out. GO terms analyses showed that common DEGs associated with depression-like behaviours were enriched in GO terms associated with cell periphery, cell-cell junction, cell cortex, integral component of synaptic vesicle membrane, actin cytoskeleton, and vesicle (Fig. 3b). In addition, KEGG pathway annotation results showed that common DEGs associated with depression-like behaviours mainly participate in endocrine system, immune system and nervous system (Fig. 3c). Moreover, KEGG pathway enrichment analysis showed that common DEGs associated with depression-like behaviours were enriched in KEGG pathways associated with nicotine addiction, insulin resistance, synaptic vesicle cycle, ether lipid metabolism, inflammatory mediator regulation of TRP channel, neurotrophin signalling pathway, glutamatergic synapse, Th1 and Th2 cell differentiation, and renin secretion (Fig. 3d).

### Identification of differentially expressed genes associated with resilience behaviours in amygdala

**Figure 4.**
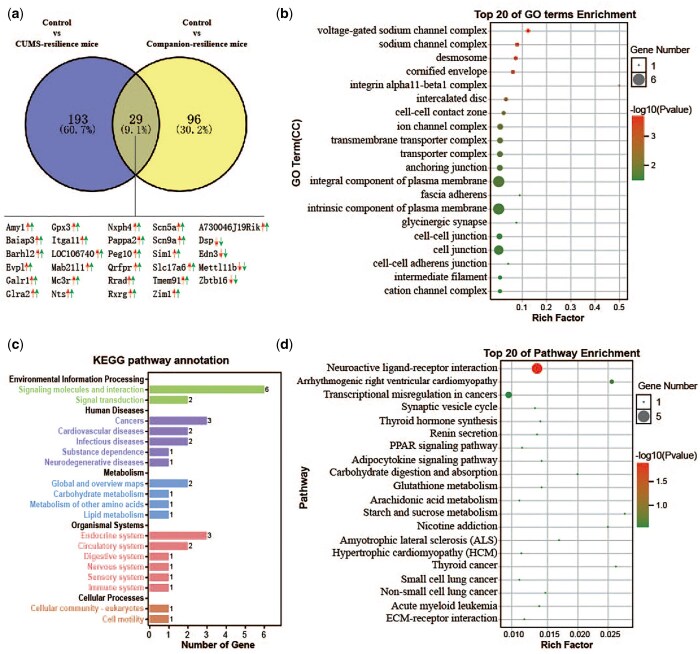
Functional analysis of DEGs associated with resilience behaviours in amygdala. (a) Screening of DEGs associated with resilience behaviours in Control versus CUMS-resilience mice and Control versus Companion-resilience mice through Venn diagram. Red arrow, gene expression pattern in Control versus CUMS-resilience. Green arrow, expression pattern of gene in Control versus Companion-resilience. (b) The histogram demonstrates common DEGs associated with resilience behaviours GO enrichment results, CC (cellular component). (c) KEGG pathway annotation for common DEGs associated with resilience behaviours. (d) The histogram demonstrates common DEGs associated with resilience behaviours KEGG pathway enrichment results, top 20 of pathway enrichment were given.

Similar to susceptible mice, mice with resilience behaviours were also obtained from the CUMS and Companion groups based on behavioural characteristics. Therefore, there may be some common differentially expressed genes in resilient mice derived from CUMS and Companion treatments that are responsible for resilience behaviours. Sequentially, DEGs in Control versus CUMS-susceptible mice and Control versus Companion-susceptible comparisons were analysed and presented using a Venn diagram (Fig. 4a). As shown in Fig. 4a and Supplementary Table S9, compared to Control mice, 193 DEGs were specifically expressed in CUMS-resilience mice, as well as 96 DEGs in Companion-resilience mice, and 29 DEGs changed in both CUMS-resilience and Companion-resilience mice, such as *Amy1*, *Baiap3*, *Barhl2*, *Dsp*, *Edn3*, *Evpl*, *Galr1*, *Glra2*, *Gpx3*, *Itga11*, *LOC106740*, *Mab21l1*, *Mc3r*, *Mettl11b*, *Nts*, *Nxph4*, *Pappa2*, *Peg10*, *Qrfpr*, *Rrad*, *Rxrg*, *Scn5a*, *Scn9a*, *Sim1*, *Slc17a6*, *Tmem91*, *Zbtb16*, *Zim1, and A730046J19Rik*. In addition, compared to Control mice, the expression patterns of the 29 common DEGs were consistent in both CUMS-resilience and Companion-resilience mice, whether up- or down-regulated (Fig. 4a).

To further explore the molecular information behind these 29 common DEGs associated with resilience behaviours in amygdala, GO and KEGG pathway enrichment analysis was carried out. GO terms analysis showed that common DEGs associated with ­resilience behaviours were enriched in GO terms associated with voltage-gated sodium channel complex, sodium channel complex, desmosome, integrin alpha11-beta1 complex, ion channel complex, transmembrane transporter complex, transporter complex, anchoring junction, glycinergic synapse, cell-cell junction, and cation channel complex (Fig. 4b). In addition, KEGG pathway annotation results showed that common DEGs associated with resilience behaviours mainly participate in endocrine system and circulatory system (Fig. 4c). Moreover, KEGG pathway enrichment analysis showed that common DEGs associated with resilience behaviours were enriched in KEGG pathways associated with neuroactive ligand-receptor interaction, arrhythmogenic right ventricular cardiomyopathy, transcriptional misregulation in cancers, and synaptic vesicle cycle (Fig. 4d).

### Identification of differentially expressed genes in the amygdala between CUMS-resilience and companion-resilience mice

**Figure 5.**
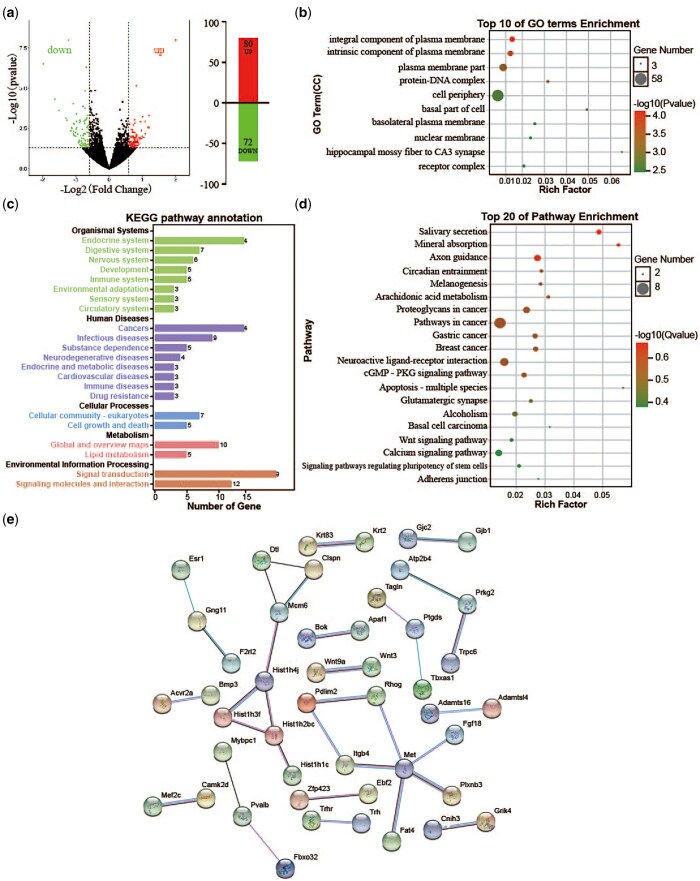
Functional analysis of DEGs associated with periodic meeting confidantes in amygdala. (a) The MA plot displays the DEGs distribution between CUMS-resilience mice and Companion-resilience mice, the column chart on the right is the number of statistical DEGs. Up represents up-regulated genes; down represents down-regulated genes. (b) The histogram demonstrates DEGs GO enrichment results, CC (cellular component). (c) KEGG pathway annotation for DEGs. (d) The histogram demonstrates DEGs KEGG pathway enrichment results, top 20 of pathway enrichment were given. (e) PPI network of the DEGs visualized by Cytoscape.

Despite some individuals in the population having long-term, persistent stress, they do not exhibit depressive-like behaviours, i.e. resilience (Southwick and Charney 2012). In our earlier studies, we also discovered mice with resilience following CUMS treatment (Si et al. 2018, Sun et al. 2018, Shen et al. 2019), and periodic meeting confidantes increased the appearance of resilience (An et al. 2020, Song and Wang 2021, Li et al. 2022). To understand the molecular changes responsible for periodic meeting confidante ameliorating CUMS-induced depression-like behaviours, DEGs associated with periodic meeting confidante treatment were screened in amygdala between CUMS-resilience and Companion-resilience mice using high-throughput sequencing. As shown in Fig. 5a and Supplementary Table S7, there were 152 DEGs in Companion-resilience mice compared to CUMS-resilience mice, with 80 mRNAs up-regulated and 72 mRNAs down-regulated.

Subsequently, GO term analysis showed that DEGs associated with periodic meeting confidantes were found to be enriched in integral component of plasma membrane, intrinsic component of plasma membrane, plasma membrane part, protein-DNA complex, cell periphery, basal part of cell, basolateral plasma membrane, nuclear membrane, hippocampal mossy fibre to CA3 synapse, and receptor complex (Fig. 5b). Furthermore, KEGG pathway annotation results showed that DEGs associated with periodic meeting confidantes mainly participated in endocrine system, digestive system and nervous system (Fig. 5c), and were enriched in axon guidance, circadian entrainment, melanogenesis, arachidonic acid metabolism, neuroactive ligand-receptor interaction, glutamatergic synapse, alcoholism, and calcium signalling pathways (Fig. 5d). Additionally, the protein-protein interactions among DEGs associated with periodic meeting confidantes were predicted by using the STRING database, *Hist1h1c*, *Hist1h2bc*, *Hist1h3f*, *Hist1h4j*, *Mcm6*, *Dtl*, and *Clspn* formed a cluster, and *Met*, *Fat4*, *Plxnb3*, *Fgf18*, *Itgb4*, *Rhog*, and *Pdlim2* formed another cluster (Fig. 5e).

### Identification of differentially expressed miRNA in the amygdala between CUMS-resilience and companion-resilience mice

**Figure 6.**
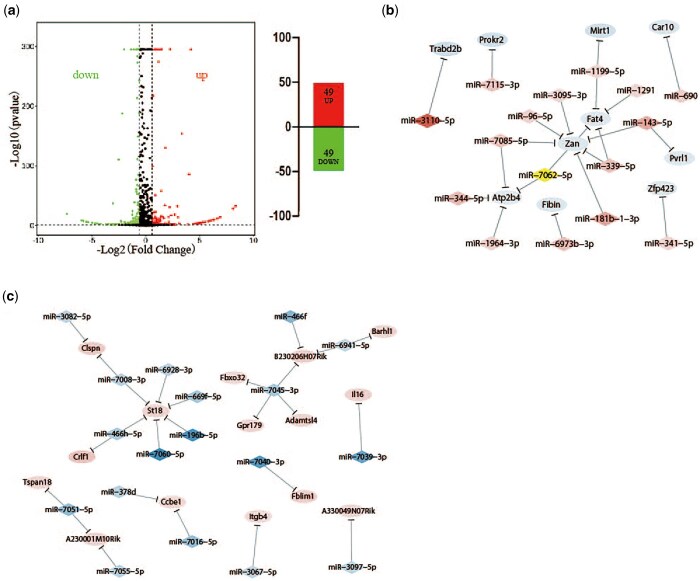
miRNA expression in resilience mice was altered after periodic meeting confidantes. (a) The MA plot displays the differently expression miRNAs distribution between CUMS-resilience mice and Companion-resilience mice, the column chart on the right is the number of statistical differently expression miRNAs. Up represents up-regulated miRNAs; down represents down-regulated miRNAs. (b, c) miRNA-mRNA network in CUMS-resilience mice versus Companion-resilience mice. The regulatory relationship was established between 33 miRNAs in miRNA profile and 25 DEGs in mRNA profile by the prediction from RNAhybrid, Targetscan, and miRanda databases. Diamonds symbols indicate miRNAs, ovals indicate mRNAs. The shades of red present the strength of the up-regulation of miRNA and mRNA. The shades of blue present the strength of the down-regulation of miRNA and mRNA.

miRNAs, which are highly conserved non-coding RNA molecules involved in gene expression regulation, have a variety of functions in psychiatric diseases (Issler and Chen 2015), and altering the brain’s levels of particular miRNAs can alter behaviour (Lin et al. 2011). To test whether periodic meeting confidante induces miRNA changes in amygdala, which in turn regulate mRNA level to ameliorate CUMS-induced depression-like behaviours, the miRNA expression profiles of amygdala in CUMS-resilience mice and Companion-resilience mice were performed by high-throughput sequencing. As shown in Fig. 6a and Supplementary Table S10, compared to CUMS-resilience mice, 98 known miRNAs were differentially expressed in Companion-resilience mice, of which 49 were up-regulated and 49 were down-regulated. In addition, the miRNA targeted genes were predicted by RNAhybrid, Targetscan and miRanda databases, and compared with DEGs associated with periodic meeting confidante in amygdala to establish the miRNA-mRNA regulation network. As shown in Fig. 6b and c, 33 miRNAs established regulatory relationships with 25 mRNAs in CUMS-resilience versus Companion-resilience mice (Supplementary Table S11).

## Discussion

It has been suggested that social relationships influence mental health (Kawachi and Berkman 2001). Depression is exacerbated by a lack of social relationships and social support, particularly low perceived emotional support (Schwarzbach et al. 2014, Santini et al. 2015). Several intervention studies have tried to improve mental health outcomes through social support, and found that social support from various sources, such as friends, colleagues, spouses, and relatives had a considerable impact on mental health (Bierman and Statland 2010, Thoits 2011). In this study, CUMS treatments led to behavioural differentiation of depression-like behaviours and resilience behaviours in mice. Periodic meeting confidantes was used as a social support treatment, and it ameliorated the CUMS-induced depression-like behaviours in mice (Fig. 1b–d). This evidence suggests that interventions aimed at increasing social support have the potential to reduce feelings of loneliness and prevent episodes of depression. However, this study exclusively utilized periodic meeting confidants of different genders to ameliorate CUMS-­induced depression-like behaviours. It is unclear whether this effect of companion on depression-like behaviours was independent of sexual desire, despite the fact that intersex in mice was prevented by regulating the duration of companion. It would be very fascinating to investigate whether meeting with confidants of the same gender also has an improvement effect on depression-like behaviour. One long-term solution to this problem could be to employ female mice as models for future same-sex companion treatment.

Over the past few decades, researchers have focused on the effects of social connections and social support on physical and mental health, and ignored the mechanisms underlying them (Cohen and Wills 1985, Berkman 1995, Cohen and Janicki-Deverts 2009, Umberson and Montez 2010). The buffering hypothesis proposed that increased social support can strengthen protective factors and/or reduce the negative effects of stressful events, which in turn promotes changes in health-related behaviours (Cohen and Wills 1985, Giesbrecht et al. 2013, Wittig et al. 2016, Leicht-Deobald et al. 2018). Affective disorders are thought to be the result of complex interactions between genetic vulnerability and adverse environmental events, once a single or multiple pathogenic factors reaches a critical level, it can result in transient or even continuous molecular and dysfunctional changes in multiple brain regions or systems, eventually leading to depression (Sullivan et al. 2000). In this study, the mRNA and miRNA profiles in the amygdala of CUMS-susceptible, Companion-susceptible, CUMS-resilience, and Companion-resilience mice were analysed using high-throughput sequencing, and genes associated with depression-like behaviours, resilience behaviours, and periodic meeting confidantes were identified.

In the mice model, we created molecular network models that dramatically improve our understanding of transcriptional mechanisms underlying depression-like behaviours by analyzing the entire transcriptome in amygdala in both CUMS-susceptible and Companion-susceptible mice. In previous studies, *Bagot et al.* performed RNA sequencing on four brain regions from Control mice and those susceptible to chronic social defeat (Bagot et al. 2016). 86 of DEGs in the amygdala overlapped with our findings in Control versus CUMS-susceptible mice (Supplementary Table S7, highlighted in yellow), including *Adam33*, *Adcyap1*, *Adora2a*, *Aldh3b2*, *Ankk1*, *Arhgap36*, *AW551984*, *Baiap3*, *Cartpt*, *Cd4*, *Cldn2*, *Col8a1*, *Col8a2*, *Cyp2s1*, *Drd2*, *Enpp2*, *Epha8*, *F5*, *Fabp7*, *Fbxo32*, *Folr1*, *Gabrq*, *Galr1*, *Glra2*, *Glra3*, *Gpr101*, *Gpr6*, *Gpr88*, *Gpx3*, *H2-Q2*, *Hap1*, *Ido1*, *Inf2*, *Kcnk9*, *Kl*, *Lrrc10b*, *Mafa*, *Mme*, *Mpp7*, *Musk*, *Nexn*, *Nts*, *Olfr1393*, *Onecut2*, *Otx2*, *Pcdh8*, *Pcp4l1*, *Pcsk1*, *Pde10a*, *Peg10*, *Plcz1*, *Ppp1r1b*, *Prima1*, *Prkcd*, *Prkch*, *Ptprv*, *Qrfpr*, *Rasgrp2*, *Rgs9*, *Ror1*, *Rrad*, *Rreb1*, *Scn4b*, *Scn5a*, *Sema3b*, *Sema7a*, *Sh3rf2*, *Slc13a4*, *Slc17a6*, *Slc17a8*, *Slc4a5*, *Smoc1*, *Sstr1*, *Steap1*, *Synpo2*, *Tmem200b*, *Tmem72*, *Tnfrsf8*, *Trhr*, *Unc5d*, *Vwa5b1*, *Zbtb16*, *Zbtb7c*, *Zim1*, *1500015O10Rik*, and *A230065H16Rik*, and 27 overlapped with our findings in Control versus Companion-susceptible mice (Supplementary Table S7, highlighted in yellow), including *Adam33*, *Adcyap1*, *C2cd4c*, *Col8a1*, *Enpp2*, *F5*, *Fabp7*, *Fam84b*, *Fbxo32*, *Gprin3*, *Lhx5*, *Nkain3*, *Onecut2*, *Pitx2*, *Prima1*, *Prkcd*, *Qrfpr*, *Rbp4*, *Rrad*, *Serpina3g*, *Slc17a6*, *Slc17a8*, *Strn*, *Tmem72*, *Tppp3*, *Trp53i11, and Trpm3.* Similarly, compared DEGs with another research that also examined the transcriptome in the amygdala of susceptible mice (Bagot et al. 2017), 33 overlapped genes were found in Control versus CUMS-susceptible mice (Supplementary Table S7, highlighted in bold font), such as *A730046J19Rik*, *Adora2a*, *Ankk1*, *Cd4*, *Chat*, *Ctxn3*, *Drd2*, *Ephb1*, *Fam83d*, *Fos*, *Gdnf*, *Gpr6*, *Gpr88*, *Hif3a*, *Isl1*, *Itga11*, *Kcna5*, *Krt2*, *Lrrc10b*, *Mep1a*, *Mid1*, *Ntrk1*, *Olfr1393*, *P2ry1*, *Paqr5*, *Ppp1r1b*, *Rbm20*, *Rgs9*, *Scn4b*, *Sfrp5*, *Sh3rf2*, *Slc10a4*, and *Thbs1*, and 18 overlapped genes in Control versus Companion-susceptible mice (Supplementary Table S7, highlighted in bold font), including *A730046J19Rik*, *Chek2*, *Ctxn3*, *Dcn*, *Ephb1*, *Fam83d*, *Fos*, *Gbx1*, *Gbx2*, *Gm4070*, *Hif3a*, *Isl1*, *Itga11*, *Ntrk1*, *Paqr5*, *Serpina3g*, *Sfrp5*, and *Slc10a4*. In cases, Daskalakis et al. have undertaken a genome-wide transcriptome, methylomic, and proteomic investigation of the subregions of the frontal cortex (PFC) and central nucleus of amygdala (CeA) in MDD using novel, large, and well-characterized post-mortem samples from MDD cases and neurologically normal controls (NCs) (Daskalakis et al. 2024). In their transcriptome screens, a total of 18 genes (Supplementary Table S7, highlighted in italic font), including *Angptl2*, *B4galt1*, *Col18a1*, *Il1r1*, *Magt1*, *Map3k6*, *Nfkbia*, *Ppp1r3b*, *Rreb1*, *Sema7a*, *Slc2a1*, *Spsb1*, *Stc2*, *Tead2*, *Thbs1*, *Tor4a*, *Trip10*, and *Zbtb7b*, were overlapped with our transcript results in Control versus CUMS-susceptible mice, and *Cd14*, *Clic4*, *Dock5*, *Iqcc*, *Map3k6*, *Mthfd2*, *Nfkbia*, *Ppp1r3b*, *Slc2a1*, *Tmbim1*, and *Vasn* were overlapped in Control versus Companion-susceptible mice (Supplementary Table S7, highlighted in italic font). What is more, DEGs identified in our research, including *Ankk1*, *Anln*, *C1qtnf2*, *C4b*, *Cd14*, *Chd7*, *Ctnna3*, *Drd2*, *Esrrg*, *Fndc5*, *Id1*, *Id3*, *Igf2*, *Itih3*, *Kcnj13*, *Map3k6*, *Mgp*, *Mid1*, *Otx2*, *Pcdha5*, *Pcdha6*, *Pgbd1*, *Plcl1*, *Prkch*, *Samd5*, *Scn4b*, *Sfrp2*, *Sim1*, *Slc25a13*, *Slc4a9*, *Smoc1*, *Snx33*, *Stard5*, *Trhr*, *Ttc12*, *Ucp2*, and *Vwa7*, were also found in previous studies derived from transcriptome, genome-wide association analysis and clinical medicine researches (Lori et al. 2018, Wray et al. 2018, Howard et al. 2019, Jaffe et al. 2022, Wingo et al. 2022, Meng et al. 2024). However, while these studies shared some molecular changes, there were also substantial differences in the molecules underlying similar behaviours, which may be due to differences in species, treatments, environments, assays, or analyses, and confirmed that depression was a heterogeneous psychiatric disease with multifactorial aetiology (Otte et al. 2016).

Human responses to stress vary greatly, with some experiencing associated psychological disturbances, others experiencing mild to moderate psychological symptoms that resolve quickly, and others not experiencing new psychological symptoms (Southwick and Charney 2012). We also obtained resilience mice derived from CUMS and periodic meeting confidante treatments, and only 29 common genes associated with resilience behaviours were obtained, with 193 genes particularly involved in CUMS-resilience mice and 96 genes particularly in Companion-resilience mice. Additionally, we compared the DEGs identified by transcriptomics screening with RNA sequencing on amygdala region from Control mice and those resilient to chronic social defeat performed previously by Bagot et al. (2017)*. B230206H07Rik*, *Car14*, *Ccbe1*, *Chrna2*, *Cyp27a1*, *Cyp2j12*, *Emilin2*, *Evpl*, *Fam83d*, *Fmo2*, *Gm13293*, *Grik4*, *Hpca*, *Inmt*, *Irx5*, *Itga11*, *Mc3r*, *Nrp1*, *Nxph2*, *Prokr2*, *Rbm20*, *Shisa6*, *Slc9a4*, *Smoc2*, *Thsd4*, *Tmem91, and Trhr* were overlapped in Control versus CUMS-resilience mice (Supplementary Table S7, highlighted in yellow), and *Adamts8*, *Bhlhe22*, *Cacng5*, *Clspn*, *Cpne5*, *Crabp1*, *Drd3*, *Ebf1*, *Evpl*, *Fam160a1*, *Fgf3*, *Fibin*, *Foxp2*, *Htr1d*, *Itga11*, *Klhl13*, *Krt2*, *Mc3r*, *Meis1*, *Mybpc1*, *Pbx3*, *Pde7b*, *Pdia5*, *Pdyn*, *Penk*, *Ptpn14*, *Ptprv*, *Rarb*, *Rgs9*, *Serpinb8*, *Slc16a3*, *Syt6*, *Tac1*, *Thbs4*, *Tmem91*, *Vgll3*, *Zfp934, and Zic2* were overlapped in Control versus Companion-resilience mice (Supplementary Table S7, highlighted in yellow). These suggested that although resilience mice derived shared the same behavioural performance, the molecules in their amygdala were quite different. In addition, common genes associated with resilience behaviours appeared to share fewer genes than common genes associated with depressive-like behaviours, and this phenomenon was also observed in the mPFC, VTA and NAc regions (An et al. 2020, Song and Wang 2021, Li et al. 2022). Therefore, compared with the molecular mechanism of stress-induced depression, the anti-stress ability displayed by individuals in the process of stress adaptation is a multidimensional construct with complex molecular mechanisms.

Behaviour is generally defined as an organism’s response to external or internal stimuli, which is largely determined by the coordinated activity of cells within the nervous system and is accompanied by numerous molecular-level changes in the brain (Sinha et al. 2020). Therefore, a large number of molecular changes in the brain may be responsible for periodic meeting confidante in ameliorating depressive-like behaviours. In this study, 152 mRNAs were obtained in CUMS-resilience versus Companion-resilience mice by high-throughput screening, which also included genes related to the nervous system, synapses, and neuroactive ligand-receptor interaction. However, because of the complexity of human behavioural aspects such as intelligence, language, personality, and emotion, genetic control of behaviour has proven more difficult to characterize in humans than in other organisms. Therefore, the importance of these genes in the prevention and treatment of depression needs further research from psychosociology, developmental sciences, neurobiology, and genetics.

Previous studies have indicated the involvement of miRNAs in the occurrences of depression or resilience relevant to chronic stress (Mouillet-Richard et al. 2012, Geaghan and Cairns 2015, Ortega et al. 2021). Although one or a few miRNAs were presumably involved in depression or resilience in response to chronic stress, these studies indicated primary data about the role of miRNA in major depression and resilience. Some of them were also found by our analysis with high throughput sequencing in CUMS-resilience versus Companion-resilience (Supplementary Table S10, highlighted in yellow), including let-7a (Smalheiser et al. 2011, Bai et al. 2014), miR-133b (Miao et al. 2018, Pei et al. 2023), miR-135a (Hu et al. 2014, Issler et al. 2014, Gheysarzadeh et al. 2018, Le Francois et al. 2018, Ding et al. 2021), miR-212 (Cao et al. 2013), miR-24 (Lopez et al. 2017), miR-301a (Smalheiser et al. 2012), miR-532 (Cao et al. 2013), and miR-96 (Sun et al. 2020). For instance, anhedonia was associated with the dysregulation of HTR4 and microRNA Let-7a in rats (Bai et al. 2014), miR-133b increased in the hippocampus of mice after fluoxetine treatment (Miao et al. 2018), miRNA-135 was essential for chronic stress resiliency, antidepressant efficacy, and intact serotonergic activity (Issler et al. 2014). Our study with high-throughput sequencing detected changes of Let-7a, miRNA-133b and miRNA-135 in the amygdala after periodic meeting confidante treatment, supporting previous data. In addition, 33 miRNAs and 25 mRNAs have been used to build the miRNA/mRNA network, which may provide a resilience-informed strategy and interventions for prevention and treatment of depression by modifying the miRNA/mRNA regulatory network.

Taken together, we discovered that periodic meeting confidantes improved CUMS-induced depression-like behaviours, with changes of mRNA and miRNA expression profiles in amygdala. Although this study did not build any causal link between gene expressions and behavioural phenotypes, it provided at least one potential intervention method and some potential drug targets for the prevention and treatment of depression.

## Supplementary Material

nsaf054_Supplementary_Data

## Data Availability

Data available on request.
