# Supplementary material for: Molecular profiles in amygdala relevant to the relief of chronic unpredicted mild stress-induced depression by periodic meeting confidantes
Source: Soc Cogn Affect Neurosci. 2025 May 23;20(1):nsaf054. doi: 10.1093/scan/nsaf054 (PMC12341916; doi:10.1093/scan/nsaf054)
Supplement: nsaf054_Supplementary_Data [file nsaf054_supplementary_data.zip › scan-24-043-File012.docx]

**Table S1. Process for Chronic Unpredictable Mild Stress and Companion treatments**

| Week | Day | CUMS and Companion treatments | Light Exposure Time |
| --- | --- | --- | --- |
| week1 | Day1 | 21:00 to next 10:00 Strobe Light, 21:00 - next 10:00 Empty Cage | 11:00-21:00 |
|  | Day2 | 13:00-14:00 Restraint, Disruption of Day-Night Rhythm | 7:00-19:00 |
|  | Day3 | 8:00-8:30 Companion, 9:00-14:00 Damp Cage, 15:30-18:30 Restraint | 11:00-19:00 |
|  | Day4 | 11:00-15:00 Noise, 16:00-22:00 Damp Cage | 0:00-12:00 |
|  | Day5 | 9:30-11:30 Restraint, 14:00-21:00 Empty Cage, 23:00-next 9:00 Strobe Light | 4:00-22:00 |
|  | Day6 | 9:00-9:30 Companion, 10:00-16:00 Damp Cage | 12:00-18:00 |
|  | Day7 | 9:00-22:00 Empty Cage, 22:00-next 14:00 Inclined Cage | 19:00-next 5:00 |
| Week2 | Day1 | 15:00-16:00 Restraint, 15:00-20:00 Noise | 21:00-next 4:00 |
|  | Day2 | 9:00-9:30 Companion, 14:00-16:00 Restraint, 20:00 - next 8:00 Strobe Light, 20:00 - next 9:00 Inclined Cage | 10:00-17:30 |
|  | Day3 | 12:00-14:00 Restraint, 18:30-23:30 Noise | 8:00-20:00 |
|  | Day4 | 10:00-20:00 Empty Cage with Water | 22:00-next3:00 |
|  | Day5 | 9:00-10:00 Restraint, 15:00-15:30 Companion, 22:00-next 10:00 Strobe Light, 22:00 - next 3:00 Noise | 14:00-20:00 |
|  | Day6 | 9:30-18:00 Damp Cage | 6:00-17:00 |
|  | Day7 | 9:00-14:00 Noise, 22:00 - next 13:00 Empty Cage | 19:00-next 7:00 |
| Week3 | Day1 | 10:00-10:30 Companion, 13:00-19:00 Noise, 21:00 - next 11:00 Empty Cage, Strobe Light | 3:00-13:00 |
|  | Day2 | 13:00-15:00 Restraint | 14:00-22:00 |
|  | Day3 | 13:00-21:30 Damp Cage, 15:30-21:30 Strobe Light | 0:00-8:00 |
|  | Day4 | 9:00-17:00 Empty Cage with Water, 19:00-19:30 Companion, 20:00 - next 2:00 Noise | 23:00-next 13:00 |
|  | Day5 | 10:00-13:00 Restraint, 21:0 0- next 9:00 Inclined Cage with Water, Strobe Light |  |
|  | Day6 | 10:30-16:30 Noise | 5:00-12:00 |
|  | Day7 | 8:30-20:30 Damp Cage, 22:00-22:30 Companion | 17:00-23:00 |
| Week4 | Day1 | 12:00-15:00 Restraint, 21:00-next 11:00 Empty Cage ＆Strobe Light | 6:00-21:00 |
|  | Day2 | 13:00-15:00 Restraint, 9:30-16:30 Noise | 7:00-19:00 |
|  | Day3 | 10:00-10:30 Companion, 11:30-21:30 Damp Cage, 10:30-21:30 Strobe Light | 11:00-19:00 |
|  | Day4 | 9:00-19:00 Empty Cage with Water, 10:00-19:00 Noise | 0:00-12:00 |
|  | Day5 | 10:00-13:00 Restraint, 21:00 - next 9:00 Inclined Cage with Water, Strobe Light | 4:00-22:00 |
|  | Day6 | 8:30-15:30 Noise, 19:00-19:30 Companion | 12:00-18:00 |
|  | Day7 | 9:00-12:00 Restraint, 12:00-24:00 Strobe Light | 17:00-23:00 |
