# Supplementary material for: Molecular profiles in amygdala relevant to the relief of chronic unpredicted mild stress-induced depression by periodic meeting confidantes
Source: Soc Cogn Affect Neurosci. 2025 May 23;20(1):nsaf054. doi: 10.1093/scan/nsaf054 (PMC12341916; doi:10.1093/scan/nsaf054)
Supplement: nsaf054_Supplementary_Data [file nsaf054_supplementary_data.zip › scan-24-043-File013.docx]

**Table S2. qRT-PCR prime information**

| **Gene ID** | **Symbol** | **Prime sequence** | **Lengths** | **Tm (°C）** |
| --- | --- | --- | --- | --- |
| 11540 | Adora2a | Forward 5′-CTCACGCAGAGTTCCATCTTCAG-3′ | 142 | 60 |
|  |  | Reverse 5′-CGAATGACAGCACCCAGCAAATC-3′ |  |  |
| 108058 | Camk2d | Forward 5′-GCTGATGCCAGTCATTGTATACAAC-3' | 124 | 60 |
|  |  | Reverse 5'-CTGCTCCTTTGGACTTGCTAGC-3' |  |  |
| 110902 | Chrna2 | Forward 5′-CCAACACTTCCGATGTGGTCATCG-3′ | 164 | 60 |
|  |  | Reverse 5'-GGAGGTGATGTTGCCAAACTCAGC-3' |  |  |
| 11438 | Chrna4 | Forward 5′-GATGATGACGACCAACGTGTGG-3' | 175 | 60 |
|  |  | Reverse 5'-GGTTAGGTGGGTGACTGCAAAG-3' |  |  |
| 11440 | Chrna6 | Forward 5'- CAATCACGCAACTGGCCAATGTG-3' | 126 | 60 |
|  |  | Reverse 5'-GTGTCTCGATGCCATCATACTCC-3' |  |  |
| 18417 | Cldn11 | Forward 5′-CTGGTGGACATCCTCATCCTTCC-3′ | 106 | 60 |
|  |  | Reverse 5'-GAACTGTCAACAGCAGCAAGATGGC-3' |  |  |
| 76441 | Daam2 | Forward 5′-CAGCAAGAGGAAGGAGCAGGAG-3' | 133 | 60 |
|  |  | Reverse 5'-CCTCTTGTCTGTCTCCTCATCTTC-3' |  |  |
| 13488 | Drd1 | Forward 5′-GAGAGCAGAGCGAGCGGCACAG-3' | 154 | 60 |
|  |  | Reverse 5'-GGGATGCTGCCTCTTCTTCTGAGAC-3' |  |  |
| 13489 | Drd2 | Forward 5′-CACCACCAACTACCTGATAGTCAG-3′ | 203 | 60 |
|  |  | Reverse 5′-CTGTGTACCTGTCGATGCTGATG-3′ |  |  |
| 77767 | Ermn | Forward 5′-GATCCTCAATGAAAACCCAGAAGAG-3′ | 149 | 60 |
|  |  | Reverse 5'-CTTCCAGGTTACTGCTGTTCGG-3' |  |  |
| 14395 | Gabra2 | Forward 5′-CCATGAGGCTTACAGTCCAAGC-3′ | 147 | 60 |
|  |  | Reverse 5′-CGGAGTCAGAAGCATTGTAAGTCC-3′ |  |  |
| 110886 | Gabra5 | Forward 5′-GATGATGGCACACTTCTCTACACC-3' | 169 | 60 |
|  |  | Reverse 5'-CAGACTTGGTGGAGCCATTGGTC-3' |  |  |
| 57249 | Gabrq | Forward 5'- GGTGCTGTCAACATATGATGTCCG-3' | 158 | 60 |
|  |  | Reverse 5'-GTGTGTCCTTCCAGGTCTGATG-3' |  |  |
| 237213 | Glra2 | Forward 5′-CAGCTGGAGAGTTTTGGGTACAC-3' | 176 | 60 |
|  |  | Reverse 5'-TTGACCTCAATGCAGGTAAACTTGC-3' |  |  |
| 14706 | Gng4 | Forward 5′-CTGTACCACCTCTCAGCAGGGAGTG-3′ | 166 | 60 |
|  |  | Reverse 5'-CACAGTAGGCCAGGAGGTCTGAG-3' |  |  |
| 110637 | Grik4 | Forward 5′-GACAAGACAGCTACTATCATCATCC-3' | 154 | 60 |
|  |  | Reverse 5'-CATCCACAAGGCTGTCCATTCTC-3' |  |  |
| 15114 | Hap1 | Forward 5′-GCTCAGGCTTCTGGAGGAAGAG-3' | 154 | 60 |
|  |  | Reverse 5'-CACCAGCACTTCCGATAGCTCTG-3' |  |  |
| 15552 | Htr1d | Forward 5′-CCAGCAGGAAACTGTACTCATTCC-3' | 144 | 60 |
|  |  | Reverse 5'-GGAGGCAAAACAAATGGGTCACC-3' |  |  |
| 16875 | Lhx8 | Forward 5′-CCTCCTCAAGGTGAATGACTTATGC-3' | 187 | 60 |
|  |  | Reverse 5'-CCAGTCAGTCGAGTGGATGTGC-3' |  |  |
| 338367 | Myo1d | Forward 5′-GTCTCGTGTGATTGTGCAGCAGC-3′ | 175 | 60 |
|  |  | Reverse 5′-CTCAGCAGCGTCATTGATGGAAGAC-3′ |  |  |
| 18013 | Neurod2 | Forward 5′-CTCTCCCCTTGACTCCTCTCTGAG-3' | 126 | 60 |
|  |  | Reverse 5'-CTTGTCGCTCCTCGGCTCGTC-3' |  |  |
| 18186 | Nrp1 | Forward 5′-CCTGGAGCAAGACTCGAATCCTC-3' | 154 | 60 |
|  |  | Reverse 5'-GGATAGAACGCCTGAAGAGGAGC-3' |  |  |
| 171171 | Ntng2 | Forward 5′-CACGGCTCTCCCAATGCCTGTG-3' | 145 | 60 |
|  |  | Reverse 5'-GGACAGCGACAGAGATAGCTACAGC-3' |  |  |
| 18211 | Ntrk1 | Forward 5'-CAGTCACCTGAATCTGTCCTCC-3' | 113 | 60 |
|  |  | Reverse 5'-CACAGGAACAATGCAGTGGGTTC-3' |  |  |
| 67405 | Nts | Forward 5′-GGAATGAATCTCCAGCTGGTGTG-3' | 161 | 60 |
|  |  | Reverse 5'-GTCATTTTCCAAGACGGAGGACTTG-3' |  |  |
| 227120 | Plcl1 | Forward 5′-GGTCCTCATAGAAATGGCTGACAC-3' | 199 | 60 |
|  |  | Reverse 5'-CTTGGCATTCTTCAGCAGGTCTC-3' |  |  |
| 19739 | Rgs9 | Forward 5′-CCAGCTGAAGACACAGACTACG-3' | 151 | 60 |
|  |  | Reverse 5'-GCTCTTTGGCCTGCATGATGAC-3' |  |  |
| 20271 | Scn5a | Forward 5′-GTGACTGGAGAGTTGTACTGGAC-3' | 173 | 60 |
|  |  | Reverse 5'-ATGTAGAGGTTGTCCTCCCACTG-3' |  |  |
| 14664 | Slc6a9 | Forward 5′-CCATACCTCTGCTATCGCAACGG-3' | 191 | 60 |
|  |  | Reverse 5'-GTGGACACCACCATCATACCATAGC-3' |  |  |
| 20496 | Slc12a2 | Forward 5'- GATTCGCAGAGACTGTGGTGGAG-3' | 153 | 60 |
|  |  | Reverse 5'-CAATCTGAGCCTTTGCTTCCCACTC-3' |  |  |
| 140919 | Slc17a6 | Forward 5′-GAGAGGAGTAGGTTGGCTACAAC-3′ | 140 | 60 |
|  |  | Reverse 5′-CAGACCATGCCAAAGCTTCCATAC-3′ |  |  |
| 216227 | Slc17a8 | Forward 5′-CTGTGTCATGGGTGTGAGGATTTTG-3′ | 126 | 60 |
|  |  | Reverse 5′-CAGAAGGAAGTCGTGGCTAGAC-3′ |  |  |
| 76376 | Slc24a2 | Forward 5′-GCATGGGTCCTGCATAACGAATG-3' | 159 | 60 |
|  |  | Reverse 5'-TTGTGGAGGGAGGCAGAGCTTC-3' |  |  |
| 20980 | Syt2 | Forward 5′-GAACGCCATGAACATGAAGGACATG-3' | 157 | 60 |
|  |  | Reverse 5'-CTGGTTGGCCTGGAAATCATAGTC-3' |  |  |
| 54524 | Syt6 | Forward 5′-GAAGGACATCCAGTACGCTACTAG-3' | 174 | 60 |
|  |  | Reverse 5'-CACAGTAGGGATACTTTGACGTAGG-3' |  |  |
| 2828187 | Gapdh | Forward 5′-CGTCCCGTAGACAAAATGGT-3′ | 110 | 60 |
|  |  | Reverse 5′-TTGATGGCAACAATCTCCAC-3′ |  |  |
