# Supplementary material for: Molecular profiles in amygdala relevant to the relief of chronic unpredicted mild stress-induced depression by periodic meeting confidantes
Source: Soc Cogn Affect Neurosci. 2025 May 23;20(1):nsaf054. doi: 10.1093/scan/nsaf054 (PMC12341916; doi:10.1093/scan/nsaf054)
Supplement: nsaf054_Supplementary_Data [file nsaf054_supplementary_data.zip › scan-24-043-File014.docx]

**Table S3. Double-tailed analysis of Sucrose preference test (SPT) in Figure 1.**

| Period of comparison | Multiple comparisons | Mean Difference. | Significant? | Summary | Adjusted P-value |
| --- | --- | --- | --- | --- | --- |
| Before CUMS | Control vs CUMS | 0.2947 | No | ns | 0.9942 |
|  | Control vs Companion | 1.126 | No | ns | 0.9189 |
|  | CUMS vs Companion | 0.8313 | No | ns | 0.9549 |
| After CUMS | Control vs CUMS | 12.06 | Yes | *** | 0.0002 |
|  | Control vs Companion | 5.077 | No | ns | 0.1870 |
|  | CUMS vs Companion | -6.987 | Yes | * | 0.0447 |
| Before CUMS-  After CUMS | Control | 1.781 | No | ns | 0.9008 |
|  | CUMS | 13.55 | Yes | *** | <0.0001 |
|  | Companion | 5.731 | No | ns | 0.1407 |

Note. Three asterisks show p < 0.001, two asterisks show p < 0.01, one asterisk show p < 0.05, in which two-way ANOVA was used for the comparisons among control group, CUMS group, and CUMS-Confidant group, and paired t-test was used for analysis of before versus after values within groups.
