# Supplementary material for: Molecular profiles in amygdala relevant to the relief of chronic unpredicted mild stress-induced depression by periodic meeting confidantes
Source: Soc Cogn Affect Neurosci. 2025 May 23;20(1):nsaf054. doi: 10.1093/scan/nsaf054 (PMC12341916; doi:10.1093/scan/nsaf054)
Supplement: nsaf054_Supplementary_Data [file nsaf054_supplementary_data.zip › scan-24-043-File015.docx]

**Table S4. Double-tailed analysis of Y-maze test (YMT) in Figure 1.**

| Period of comparison | Multiple comparisons | Mean Difference. | Significant? | Summary | Adjusted P-value |
| --- | --- | --- | --- | --- | --- |
| Before CUMS | Control vs CUMS | 0.9247 | No | ns | 0.9885 |
|  | Control vs Companion | -1.546 | No | ns | 0.9500 |
|  | CUMS vs Companion | -2.471 | No | ns | 0.8282 |
| After CUMS | Control vs CUMS | 15.11 | Yes | **** | ＜0.0001 |
|  | Control vs Companion | 7.211 | No | ns | 0.0801 |
|  | CUMS vs Companion | -7.896 | Yes | * | 0.0474 |
| Before CUMS-  After CUMS | Control | -0.912 | No | ns | 0.9889 |
|  | CUMS | 13.27 | Yes | *** | 0.0003 |
|  | Companion | 7.845 | Yes | * | 0.0493 |

Note. Four asterisks show p < 0.0001, Three asterisks show p < 0.001, two asterisks show p < 0.01, in which two-way ANOVA was used for the comparisons among control group, CUMS group, and CUMS-Confidant group, and paired t-test was used for analysis of before versus after values within groups.
