# Supplementary material for: Molecular profiles in amygdala relevant to the relief of chronic unpredicted mild stress-induced depression by periodic meeting confidantes
Source: Soc Cogn Affect Neurosci. 2025 May 23;20(1):nsaf054. doi: 10.1093/scan/nsaf054 (PMC12341916; doi:10.1093/scan/nsaf054)
Supplement: nsaf054_Supplementary_Data [file nsaf054_supplementary_data.zip › scan-24-043-File016.docx]

**Table S5.** **Double-tailed analysis of Forced swimming test (FST) in Figure 1.**

| Period of comparison | Multiple comparisons | Mean Difference. | Significant? | Summary | Adjusted P-value |
| --- | --- | --- | --- | --- | --- |
| Before CUMS | Control vs CUMS | 4.673 | No | ns | 0.9117 |
|  | Control vs Companion | 2.629 | No | ns | 0.9711 |
|  | CUMS vs Companion | -2.044 | No | ns | 0.9824 |
| After CUMS | Control vs CUMS | -42.84 | Yes | *** | 0.0009 |
|  | Control vs Companion | -15.59 | No | ns | 0.3624 |
|  | CUMS vs Companion | 27.25 | Yes | * | 0.0496 |
| Before CUMS-  After CUMS | Control | 3.771 | No | ns | 0.9014 |
|  | CUMS | -43.74 | Yes | *** | 0.0004 |
|  | Companion | -14.45 | No | ns | 0.4723 |

Note. Three asterisks show p < 0.001, one asterisk show p < 0.05, in which two-way ANOVA was used for the comparisons among control group, CUMS group, and CUMS-Confidant group, and paired t-test was used for analysis of before versus after values within groups.
