# Supplementary material for: Molecular profiles in amygdala relevant to the relief of chronic unpredicted mild stress-induced depression by periodic meeting confidantes
Source: Soc Cogn Affect Neurosci. 2025 May 23;20(1):nsaf054. doi: 10.1093/scan/nsaf054 (PMC12341916; doi:10.1093/scan/nsaf054)
Supplement: nsaf054_Supplementary_Data [file nsaf054_supplementary_data.zip › scan-24-043-File017.docx]

**Table S6.** **Proportion for susceptible and resilient mice in the group of CUMS and Companion.**

| **Mice** | **CUMS** | **Percentage (%)** | **CUMS-companion** | **Percentages (%)** |
| --- | --- | --- | --- | --- |
| susceptible | 5 | 33.33 | 3 | 20.00 |
| resilient | 2 | 13.33 | 5 | 33.33 |
| Atypical | 8 | 53.33 | 7 | 46.67 |
| Total | 15 | 100 | 15 | 100 |
